# Genotyping of Acanthamoeba Isolated From Surface and Stagnant Waters of Qazvin, Central Iran

**DOI:** 10.5812/ircmj.4121

**Published:** 2013-06-05

**Authors:** Hossein Hooshyar, Bahram Hosseinbigi, Mehrzad Saraei, Safarali Alizadeh, Mohammad Eftakhar, Sima Rasti, Nader Khosro-Shahi

**Affiliations:** 1 Department of Parasitology, School of Medicine, Kashan University of Medical Sciences, Kashan, IR Iran; 2 Department of Parasitology, School of Medicine, Qazvin University of Medical Sciences, Qazvin, IR Iran; 3 Department of Microbiology, School of Public Health, Tehran University of Medical Sciences, Tehran, IR Iran

**Keywords:** Amoeba, Acanthamoeba, Iran

## Dear Editor,

The free living amoeba, Acanthamoeba spp are found and distributed in a wide variety of environmental sources including air, water, soil dust, vegetables as well as the skin, cornea, lung and brain of human (1). Isolation of Acanthamoeba cyst from environmental sources such as soil, fresh surface water and stagnant water have been showed that this environmental sources have potential importance for transmission of this amoeba in human and others mammals (2). The resistant double-layered coat cyst of this amoeba can survive in water in the presence of chlorine and other disinfectants.

Several species of Acanthamoeba have been known to cause life-threatening granulomatus amoebic encephalitis (GAE), pneumonitis, skin serious lesion in immune compromised individuals, and amoebic keratitis in contact lens wearers. Some studies showed that the incidence of human Acanthamoeba infections especially amoebic keratitis increased through worlds during that last 30 years (1). The identification of Acanthamoeba can be easily accomplished by the morphological characteristic of cysts; however the morphological feature of cysts can be change with the culture condition and be variable within the same strain (1). The most recently proposed method for molecular identification and taxonomy of Acanthamoeba is sequence analysis of variable region of 18s rRNA gene. To date, based on 18s rRNA sequencing, the Acanthamoeba genus classified into 17 (T1-T17) different genotypes (1). Studies showed T4 genotype of Acanthamoeba is most important causative agent of amoebic keratitis and GAE. In Iran, genotypes of T2, T4, T6 were previously reported from some environmental sources such as fresh and stagnant water, soil and hot water (3-5). Since few studies regarding Acanthamoeba genotypes isolated from stagnant water have been previously reported in Iran and important of it to separate of Acanthamoeba cysts, This study was conducted to determine the presence and identify Acanthamoeba in stagnant water of Qazvin, central Iran, using culture and molecular methods.

This study was conducted as a descriptive cross-sectional study. From September to December 2010 a total of 40 water samples were collected randomly from 20 stagnant water of square and parks in different region of Qazvin. All samples put in to 250-400 ml plastic bottles and were carried to Parasitology laboratory in Qazvin University of medical sciences. Water samples were filtered using sterile cellulose nitrate membrane filter (Millipore, Pore size 0.45 µm). Each filter was separated and cultured on 1.5% non-nutrient agar medium was prepared with amoeba page saline that overlaid with heat-killed Escherichia coli (5). The plates were incubated at 25-30 ˚^c^ and monitored for present of trophozoite or cyst of amoeba daily until 30 days. The trophozoites and cyst were harvested by phosphate buffered saline (PBS) and using a cell scraper. For washing, centrifugation of amoeba in PBS (PH = 7.2) three times at 4000 rpm for 5 minute was performed. The supernatants were then discarded and cell pellets were re-suspended in DNG lysis buffer and DNA extraction was performed by DNGTM – PLUS kit (cinna gen, Iran). PCR reaction was performed using a pair of primer (JDP1-JDP2) that amplified a fragment of 423 to 551 bp of Acanthamoeba specific 18s rDNA (6). The sequence of primer in this study were:

(5́-GGCCCAGATCGTTTACCGTGAA) as forward.

(5́-TCTCACAAGCTGCTAGGGGAGTCA) as reverse.

The PCR reaction was carried out in 25µl cotaining 1.25 U Taq DNA Polymerase, 20 ng DNA, 1.5 Mm Mgcl2, 300 µl dNTP and 0.2 µg of each primers. The amplification was done in a PCR machine, Veriti Thermal Cycler (Applied Biosystems, USA) as following: an initial denaturing step at 94 ˚^c^ for 5 minute and 32 repetition at 94 ˚^c^ for 30 second (denaturation), 60 ˚^c^ for 30 second (annealing), 72˚^c^ for 30 second (extension) with a final extension step for 5 minute at 72˚^c^. Amplified DNA was electrophosed on 1.5% agarose gel, stained with a solution of 0.5 µg/ml of ethidium bromide and visualized under UV light.

**Figure 1.**
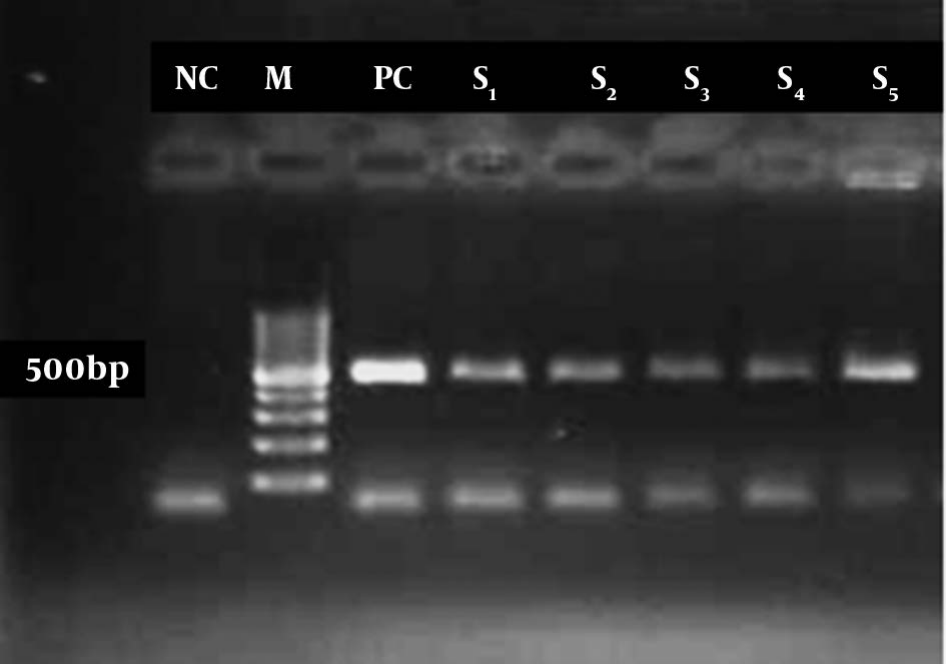
PCR product of free living amoeba from culture. M = marker, NC = negative control, PC = positive control, S = sample

PCR product were purified using DNA zol® R reagent Kit (Inviyrogen, USA) and was submitted for sequencing by using an automatic sequencer, 3130xl Genetic Analyzer (ABI.co, USA) in the reference health laboratory, Qazvin University of Medical Sciences. The results of sequencing were edited manually and analyzed by Choromas version 1.45 (Technelysium, Australia) and aligned using Genrunner ver 3.05 program. Totally 32 (80%) out of 40 water samples were positive for free living amoebas by culture method.PCR reaction with 18s rRNA gene primer were positive in 14 (43.8%) of 32 culture samples and showed a 500 base-pair approximately Acnthamoeba specific PCR product in gel electrophoresis (Figure 1). 

After purify of PCR product and eliminate primers, sequencing analysis of isolate in Gen Bank data base showed that 11 (78.6%) of positive isolates belonging to T4 genotype and three isolates (21.4%) belonged to the T2 genotype. Search by Basic Local Alignment Search Tool (blast) program in gene data banks (NCBI, NIH), showed 8 (57.2%) 0f isolates were Acanthamoeba sp, 3 (21.4%) A. palestinensis, and 3 (21.4%) A. polyphaga. This study showed Acanthamoeba is a prevalent free living amoeba in stagnant water of Qazvin. Cyst of Acanthamoeba species can withstand drying and thus transport by water and air is possible (2). Isolation of Acanthamoeba from different sources of water such as swimming pools, ponds and stagnant water showed that these waters are suitable sources for distribution of cyst in human environmental. Many clinical cases of human Keratitis due to Acanthamoeba have been reported in Iran (5) and this is revealed that there was a considerable increase in the incidence of Acanthamoeba Keratitis in Iran during the recent years for example Rezaiean et al. (7) have been diagnosed and reported 45 cases of Acanthamoeba keratitis during 1995-2005 in protozoal laboratory in Tehran university of medical sciences.

Based on 18s rRNA sequencing, most of Acanthamoeba isolated from keratitis in the world have been typed as the T4 genotype (1). Genotypic Identification of Acanthamoeba sp. Isolates Associated with an Outbreak of Acanthamoeba keratitis in Chicago-Gary-Kenosha area indicate that Acanthamoeba keratitis cases are associated predominantly (approximately 97%) with a single genotype (designated T4) of Acanthamoeba and rarely with other genotypes such as T3 and T11 (8). Previous study in Iran showed that T4 genotype was a predominant type found in Acanthamoeba keratitis patients (4, 5). Maghsood et al. (4) tested 13 Acanthamoeba keratitis cases presented during 1998–2003 at the Tehran University of Medical Sciences, Iran and find that eight (61.5 %) belonged to T4, two (15.3 %) belonged to T3 and three (23 %) belonged to the T2 genotype. T4 genotype has been reported from different environmental sources in Iran. Badrizadeh et al. (3) isolated A. castellanii (T4) from 3.6% of Sarein hot springs in Ardabil province. Study on 12 pool and waterfall samples showed 58% T2 and 33% T4 genotype (4). The T4 genotype also isolated from water in recreational areas, surface waters, hospital wards, soil and dust sources, in Iran (5, 9, 10). The present study revealed that T4 genotype of Acanthamoeba is most common isolate of Acanthamoeba in Qazvin stagnant of waters. Due to stagnant waters can be potential sources for distribution and transmission of Acanthamoeba cysts, more attention of health practitioner regarding to free living amoeba is recommended.
